# Development of Risperidone PLGA Microspheres

**DOI:** 10.1155/2014/620464

**Published:** 2014-01-28

**Authors:** Susan D'Souza, Jabar A. Faraj, Stefano Giovagnoli, Patrick P. DeLuca

**Affiliations:** ^1^Sunovion Pharmaceuticals Inc., Marlborough, MA 01752, USA; ^2^Fresenius Kabi USA, Skokie, IL 60077, USA; ^3^Department of Chemistry and Technology of Drugs, Università degli Studi di Perugia, Via del Liceo 1, 06123 Perugia, Italy; ^4^University of Kentucky College of Pharmacy, Lexington, KY 40536, USA

## Abstract

The aim of this study was to design and evaluate biodegradable PLGA microspheres for sustained delivery of Risperidone, with an eventual goal of avoiding combination therapy for the treatment of schizophrenia. Two PLGA copolymers (50 : 50 and 75 : 25) were used to prepare four microsphere formulations of Risperidone. The microspheres were characterized by several *in vitro* techniques. *In vivo* studies in male Sprague-Dawley rats at 20 and 40 mg/kg doses revealed that all formulations exhibited an initial burst followed by sustained release of the active moiety. Additionally, formulations prepared with 50 : 50 PLGA had a shorter duration of action and lower cumulative AUC levels than the 75 : 25 PLGA microspheres. A simulation of multiple dosing at weekly or 15-day regimen revealed pulsatile behavior for all formulations with steady state being achieved by the second dose. Overall, the clinical use of *Formulations A, B, C, or D* will eliminate the need for combination oral therapy and reduce time to achieve steady state, with a smaller washout period upon cessation of therapy. Results of this study prove the suitability of using PLGA copolymers of varying composition and molecular weight to develop sustained release formulations that can tailor *in vivo* behavior and enhance pharmacological effectiveness of the drug.

## 1. Background

The treatment of schizophrenia using oral conventional antipsychotics dates back to the mid-1950s. Administration of antipsychotic drugs via the oral route offered several advantages in terms of ease of administration, noninvasiveness of therapy, and portability of medication. It is common knowledge that injectable depot formulations possess a number of advantages over oral dosage forms such as avoidance of first-pass metabolism and the certainty of delivery of the therapeutic agent [1–3]. Therefore, by the 1960s, the first injectable depot conventional antipsychotic was introduced [1]. The sustained release properties of the injectable depot led to significant strides in the treatment of schizophrenia as it reduced relapse rates in comparison to the oral dosage form. A reduction in the number of days of hospitalization for patients on injectable antipsychotics over those on oral medication was also documented by researchers [4]. Despite being an injectable, it was noted that patients preferred injectable depot antipsychotics over oral agents. Additionally, the use of injectable depot preparations for the treatment of schizophrenia was considered beneficial as it ensured adherence to treatment over an extended duration leading to improved health outcomes [4–7]. Compliance with treatment regimens sharply increased when patients were switched to depot agents, allowing physicians a better mechanism to detect noncompliance to therapy. Further, the injectable depot allowed better control over drug management and more predictable and consistent plasma drug concentrations when compared with oral formulations [8]. In general, injectable depots were well tolerated and more clinically efficacious than oral preparations [4, 9].

The second generation antipsychotics or atypical antipsychotics were introduced in the 1980s and led to significant improvements in the treatment of schizophrenia. Atypicals, effective for the positive symptoms of schizophrenia, demonstrated a lack of negative symptoms leading to greater efficacy and reduced side effects. Indeed, atypical antipsychotics have a substantially better adverse effect profile than first generation antipsychotics with respect to movement disorders, akathisia, and tardive dyskinesia [10]. Notably, concerns with extrapyramidal symptoms (EPS) and the risk of tardive dyskinesia with older antipsychotics led to a reluctance in accepting injectable depots of first generation antipsychotics and a preference for oral atypical antipsychotics [11].

Risperidone, a novel benzisoxazole-type atypical antipsychotic, is effective in the treatment of positive as well as negative symptoms of schizophrenia and has a low incidence of extrapyramidal side effects [12–16]. *In vivo*, Risperidone is extensively metabolized by cytochrome P450 2D6 (subject to genetic polymorphism) to form its main metabolite, 9-hydroxyrisperidone, via hydroxylation and N-dealkylation pathways [17, 18]. 9-Hydroxyrisperidone displays similar pharmacological activity to the parent compound; thus, the active moiety *in vivo *is a summation of both species. Clinically, the efficacy of Risperidone has been well established and is effective against positive and negative symptoms of schizophrenia [19, 20]. Risperidone is an antagonist of the 5HT2A receptor compared with the D2 receptor which allows for a greater efficacy against negative symptoms and a lower rate of EPS which makes it a suitable candidate for treatment of schizophrenia [19].

Two decades of clinical usage have clearly established that atypical antipsychotics like Risperidone offer several benefits including reduced concerns with movement disorders and greater efficacy for negative and mood symptoms than first generation antipsychotics [21]. However, these benefits diminish greatly in patients who suffer from severe psychiatric ailments primarily due to non-adherence to oral therapy. Several reports have documented the reduced effectiveness of oral Risperidone therapy in young and old schizophrenic patients [22, 23]. Daily dosing of oral Risperidone is non-ideal due to patient resistance to treatment and often ineffective given that efficacy depends on constant adherence to therapy. Despite the fact that adherence to daily medication has been better in schizophrenic patients dosed with atypical antipsychotics than conventional antipsychotics, Dolder et al. recorded that poor compliance issues persisted in schizophrenic patients [24].

A critical factor in achieving beneficial long term outcomes is in establishing a mechanism wherein the schizophrenic patients demonstrate adherence to treatment cycles. Infrequent intake of medication or partial dosing is far more common than complete non-adherence to therapy posing a significant challenge to patients, caregivers, and society at large. Robinson et al. reported a five-fold increase in the risk of relapse with patients who partially adhered to treatment [25]. Incidence of relapse in schizophrenic patients carries a large economic and personal cost. Relapsed patients suffer from reversal of gains achieved during therapy, loss of function, demoralization, loss of confidence, danger to self or others, and loss of job leading to a loss in productivity and opportunity. Further, rehospitalization of relapsed patients places a huge economic burden on existing healthcare system in the US [26].

Continuous delivery of the atypical antipsychotic is an effective way to ensure adherence to therapy with minimal relapse. Analogous to the first generation antipsychotics, injectable depot formulations of Risperidone were developed and marketed. Studies on long acting Risperidone revealed its efficaciousness in the treatment of schizophrenia and schizoaffective disorder [8, 27]. Extended treatment with long acting Risperidone also reduced movement disorders relative to baseline in patients clinically stable on a variety of antipsychotic drugs [28].

However, a major drawback of the currently marketed long acting Risperidone, administered every 15 days, necessitates an additional supplementation with oral Risperidone for three weeks after administration of the injectable formulation. While challenges related to patient compliance continue to persist with oral therapy, oral supplementation is necessary due to the delayed response profile obtained with the injectable preparation where drug release occurs approximately 3 weeks after administration. Published literature cites that *in vivo* levels peak 4-5 weeks after dosing, for a 7-week duration of action [27, 29]. Co-administration of oral Risperidone, while necessary in an inpatient or outpatient setting, is inconvenient and poses major compliance issues in patients with psychotic disorders. Additionally, costs incurred with co-administration therapy of Risperidone are high [30, 31]. Thus, the latency in drug release is a major shortcoming of the long acting Risperidone depot preparation. Therefore, there is a strong need for a non-oral controlled delivery dosage form for this drug.

Over the years, several polymers have been evaluated for development of controlled release injectable formulations. Of these polymers, one class of polymers has achieved significant commercial success in the pharmaceutical market. The polylactide (PLA) and polylactide-co-glycolide (PLGA) class of polymers are biodegradable, biocompatible, and nontoxic and have a long history of use [32]. *In vivo*, they are hydrolyzed into metabolic products that are easily eliminated from the body. Initially approved for surgical use in humans by the US Food and Drug Administration, they have since been used to formulate a wide range of therapeutic agents [33, 34]. A few commercially available formulations using PLA or PLGA polymers include Lupron Depot, Somatuline LA, and Trelstar Depot [35]. These polymers have been shown to be efficacious in the delivery of biologically active agents and also improve patient compliance by eliminating the need for frequent administration [36].

PLGA polymers are well suited for controlled delivery of drugs via the parenteral route as they exhibit good mechanical properties and demonstrate predictable degradation kinetics. Notably, polymeric microspheres prepared using PLGA have been successful in ensuring sustained release of therapeutic agents for various drugs [37]. Several examples in literature discuss their effectiveness in providing targeted drug levels *in vivo*, for long periods of time [38–40]. For this reason, they are popular as delivery vehicles for drugs where sustained release is desired for extended intervals, ranging from a few weeks to several months [41, 42]. These polymers are also used in marketed injectable formulations as carriers to deliver antipsychotic drugs and are noted to provide benefits over conventional oral therapy [43]. A striking benefit of using PLGA polymers to deliver atypical antipsychotics includes a reduction in dosing frequency leading to measurable increase in adherence to treatment regimens in a schizophrenic patient population [44, 45]. In general, the success of PLGA polymers as delivery systems is due to the fact that polymer properties are well understood and can be customized to afford sustained drug release. For instance, selection of copolymers of various lactide : glycolide with variable molecular weights is an effective way to control polymer degradation rate and drug release. By changing the composition of lactide or glycolide in the copolymer, a wide range of degradation rates can be obtained. An increase in the more hydrophobic lactide moiety ensures a slower degradation rate of the PLGA polymer leading to extended duration of drug release [46]. Similarly, utilization of a higher molecular weight copolymer increases degradation times leading to prolonged drug release. Additional properties that can be varied include polymer crystallinity and glass transition temperature. These physical and chemical properties have been well studied and characterized leading to predictable degradation kinetics of the PLGA polymer, *in vitro* and/or *in vivo*.

Upon *in vivo* administration of a PLGA based injectable depot, water interacts with the polymer and hydrolysis of the ester bonds commences. As the polymer degrades, its hydrophobicity decreases and the number of hydrophilic hydroxyl and carboxylic acid end groups in the matrix increases. An accumulation of hydrophilic acidic end groups has a twofold effect: (1) it increases the amount of water incursion into the polymer and (2) initiates autocatalysis of the polymer matrix [47]. Therefore, polymer degradation and, consequently, drug release from PLGA is a very complex and dynamic process. This is of particular significance as it provides the researcher a scientifically sound approach to select an appropriate polymer specific to a therapeutic need or treatment regimen.

When plotted as a function of time, drug release from a PLGA matrix occurs in three phases [32]. The first phase of release is known as “initial burst” and occurs as a result of detachment of surface associated drug or drug that is easily dissociated from accessible pores in the polymeric microspheres. Depending on the surface area and porosity, a high or low initial burst may be observed. The second phase of release, that is, diffusional release, is a consequence of initial polymer hydration and is followed by “erosional release” or the final phase of drug release. Once the polymer is hydrated, polymer autocatalysis ensues causing bulk hydrolysis, that is, complete polymer degradation and erosion (mass loss). Previous reports have also documented that properties of the formulation have an impact on drug release kinetics [48]. Therefore, depending on the properties of the polymer and the microsphere dosage form, the rate and extent of each of these phases can be altered to customize drug release profiles. Hence, in this study, two PLGA copolymers having varying molecular weights and lactide : glycolide ratios as well as drug loading were evaluated with an aim to obtain Risperidone PLGA microspheres having varying duration of action. Results and discussions related to the findings of the study demonstrate the suitability of this approach in developing sustained release formulations where *in vivo* behavior can be customized to meet patient needs.

## 2. Materials and Methods

### 2.1. Materials

Risperidone was purchased from Cipla Ltd., India, and PLGA 50 : 50 (45 and 74 kDa) and 75 : 25 (54 and 65 kDa) from Boehringer Ingelheim (Ingelheim, Germany) and Alkermes (Cambridge, MA). All other chemicals were obtained commercially as analytical grade reagents.

### 2.2. Preparation of Microspheres

The four formulations evaluated were

- 45 kDa PLGA, 50 : 50 lactide : glycolide (*Formulation A*),
- 74 kDa PLGA, 50 : 50 lactide : glycolide (*Formulation B*),
- 54 kDa PLGA, 75 : 25 lactide : glycolide (*Formulation C*),
- 65 kDa PLGA, 75 : 25 lactide : glycolide (*Formulation D*).

Briefly, four Risperidone PLGA (*Formulations A, B, C, and D*) microspheres formulations were prepared by a solvent extraction/evaporation method [41]. Briefly, a solution of drug and polymer (10–20% polymer concentration) in dichloromethane was injected into an aqueous continuous phase at a ratio between 250 and 350 parts of polymer phase : aqueous phase, under stirring with a Silverson L4R mixer (Silverson machines, MA, USA) at 5000 rpm. Subsequently, the solvents were removed by stirring after which the microspheres were recovered by filtration, suspended in a suitable vehicle, filled into vials, and freeze-dried. The microspheres were characterized as described in Section 2.3.

### 2.3. Characterization of Microspheres

#### 2.3.1. Particle Size

Particle size distribution of the microspheres prior to vialing was determined using a laser diffraction technique (Malvern 2600c Particle Sizer, Malvern, UK). The particles were suspended in 0.05% Tween 80 and counted using a laser sensor [41]. The average particle size was expressed as volume mean diameter in microns (*μ*m).

#### 2.3.2. Surface Morphology

The surface morphology was examined by scanning electron microscopy (SEM) (Hitachi S800, Japan) at an appropriate magnification, after palladium/gold coating of the microsphere sample on an aluminum stub.

#### 2.3.3. Bulk Density

Bulk density of the microspheres was determined by transferring a weighed amount of microspheres to a graduated cylinder. The cylinder was subsequently tapped 50 times from a vertical distance of approximately 0.5 inches and the occupied volume recorded. The tapping process was repeated until the volume occupied by particles remained unchanged. The final volume was recorded as bulk volume, *V*_*b*_, and the tapped bulk density (g/cc) was calculated as *M*/*V*_*b*_, where “*M*” was the weight of microspheres employed.

#### 2.3.4. Drug Content

Risperidone content in the microspheres was analyzed by a reverse phase HPLC method using a Nucleosil C-18 column (Phenomenex, Torrance, CA) at a flow rate of 1 mL/min. The mobile phase consisted of 30% v/v acetonitrile and 0.1% (v/v) trifluoroacetic acid in water. Drug content (%) was expressed as the “weight of drug in microspheres/weight of microspheres × 100.”

#### 2.3.5. *In Vivo* Studies

In accordance with Institutional Guidelines and an in-house developed and an approved protocol, four groups of male Sprague-Dawley rats (Harlan Inc., Indianapolis, IN) weighing approximately 300 gm were used in the *in vivo* study. *Group 1* received *Formulation A*, *Group 2* received *Formulation B*, *Group 3* received *Formulation C,* and *Group 4* received *Formulation D*.

Briefly, vials containing freeze dried microspheres along with diluent were reconstituted with WFI (water for injection) and injected subcutaneously at the base of the rat neck at a dose of 20 or 40 mg/kg Risperidone (Table 1). Blood was sampled from the rat tail vein at predetermined intervals, after which the samples were centrifuged in Microtainer tubes (Becton Dickinson & Co., Franklin Lakes, NJ) and serum was collected. Serum samples for each of *Group 1 *(*Formulation A*),* Group 2* (*Formulation B*),* Group 3 (Formulation C*), and* Group 4* (*Formulation D*) were frozen and stored at −20°C until analysis. Subsequently, serum levels were assessed at Medtox Labs, USA, using a validated analytical method.

## 3. Results and Discussion

### 3.1. Polymer Selection

Properties of the four formulations used in this study are shown in Table 1. *Formulations A and B* were prepared with 50 : 50 PLGA at molecular weights 45 and 74 kDa, respectively, while *Formulations C and D* were manufactured from 54 and 65 kDa PLGA having a 75 : 25 lactide : glycolide ratio. Based on the molecular weight and copolymer ratio, *Formulations A and B* were expected to have a shorter duration of action while *Formulations C and D* would provide a more prolonged *in vivo* drug release profile due to a higher lactide content in the 75 : 25 copolymer.

#### 3.1.1. Morphology of Risperidone Microspheres

**Figure 1:**
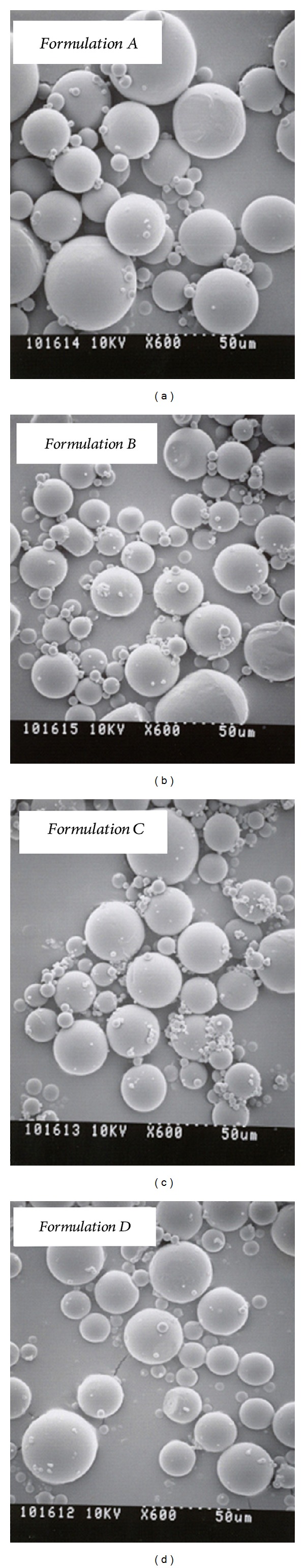
SEMs of Risperidone PLGA microspheres.

The scanning electron micrographs revealed a spherical shape with a smooth surface and homogeneous particle size distribution (Figure 1) that would be appropriate for subcutaneous administration to rats. Additionally, the microspheres could not be fractured suggesting that the interior of all four formulations was not hollow. When viewed at the same magnification (Figure 1), the particle size of *Formulation A* appeared marginally larger than *Formulation B*, while the particle size of *Formulation C* was slightly smaller than *Formulation D*. A glance at Table 1 confirms these observations as the mean particle sizes for *Formulations A*–*D* were 24.6, 18.9, 17.1, and 21.9 µm, respectively. For dosage forms like drug loaded microspheres, measurement of particle size is important as it impacts “initial burst” release [49]. A smaller particle size confers a higher surface area to volume ratio to the dosage form. It follows that a larger surface area allows for rapid water incursion and consequently, faster dissolution of drug molecules that are associated with the outer surface or accessible pores. Hence, an initial burst is expected with smaller sized microspheres.

From literature, the particle size of the commercial long acting Risperidone microsphere formulation has been reported to be between 25 and 150 *μ*m [50], significantly larger than *Formulations A, B, C, and D*. Hence, the SEM results in Figure 1 indicated that the release profiles from the four formulations would be vastly different from the marketed preparation. For instance, an “initial burst” of drug release was expected for all the formulations. Given that the particle sizes for *Formulations A*–*D* are quite similar overall, the extent of “initial burst” was expected to be broadly similar.

#### 3.1.2. Bulk Density

Bulk density values for PLGA microsphere formulations are routinely measured as they provide information on the porous network in these dosage forms. Density is inversely proportional to porosity, and a change in this parameter indicates inefficient packing due to the presence of nonspherical microspheres or the formation of hollow microspheres [51]. A relationship between bulk density, surface area, and onset of mass loss has also been reported by Mehta et al. [33]. Hence, a low bulk density is indicative of highly porous microspheres, since porosity correlates well with polymer hydration, and thereby, degradation; bulk density values are an indicator of drug release rates [52, 53].

Table 1 summarizes the results of bulk density measurements. Values for all the formulations ranged from 0.65 to 0.76 g/cc. The high bulk density values were indicative of a low degree of internal porosity with similar pore volumes for *Formulations A*–*D*. Given that particle sizes for all four formulations are similar and bulk density is high, both parameters were expected to contribute equally to the initial burst release from the microspheres.

#### 3.1.3. Drug Content

Drug content is an important property of the microsphere dosage form as it provides information related to the amount of drug available for release from the dosage form. Results of drug content, as determined by HPLC, are presented in Table 1. For the purposes of the current study, high drug loadings were targeted in part to mimic the loading level of 38.1% in the marketed Risperidone depot formulation [50]. Therefore, *Formulations A*–*D* were prepared at loadings between 25 and 34% (Table 1). These values suggest a high drug : polymer ratio for the four formulations, at a value higher than the drug solubility in the polymer. This situation favors the initial burst release phenomenon. Hence, a high value of initial burst was expected for all four formulations.

Based on the morphology, particle size, and drug content data, the formulations were expected to behave in the following manner: (a) High initial burst was expected from all the formulations, and (b) *Formulations A and B*, manufactured using 50 : 50 PLGA, were expected to exhibit a shorter duration of action than *Formulations C and D*, where the duration of action was expected to be prolonged.

### 3.2. *In Vivo* Results

#### 3.2.1. Serum Levels of Risperidone and Its Metabolite for *Formulations A, B, C, and D*

**Figure 2:**
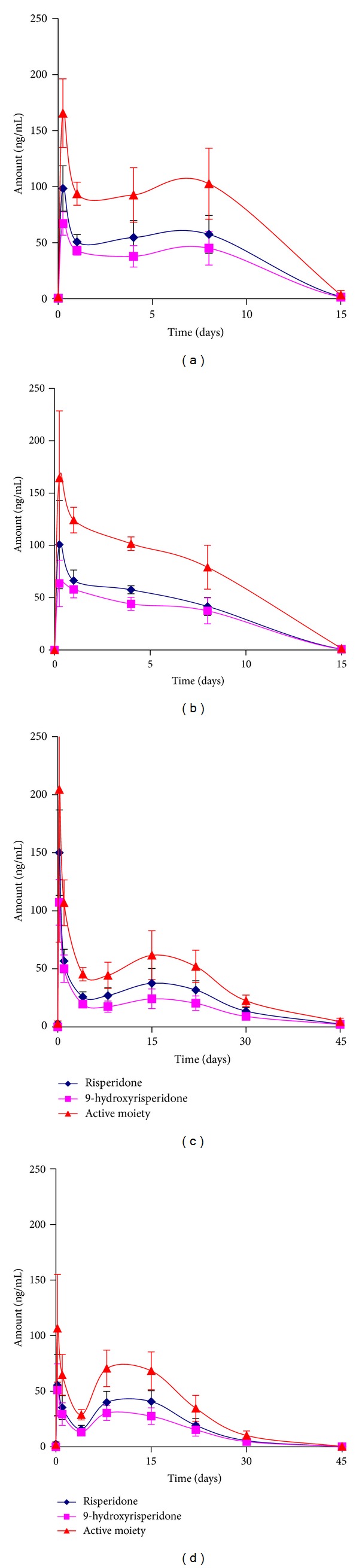
*In vivo* release of Risperidone and 9-hydroxyrisperidone from microsphere *Formulations A, B, C, and D*.

*In vivo*, Risperidone is extensively metabolized in the liver by CYP2D6 to form 9-hydroxyrisperidone, a pharmacologically active metabolite. Serum levels of Risperidone and its metabolite for each formulation, after administration of a single subcutaneous dose, are shown in Figures 2(a) to 2(d), including the levels of “active moiety” which is the sum of Risperidone and metabolite levels.

*Formulations A and B* administered to a 20 mg/kg dose in rats showed an initial burst around 100 ng/mL of Risperidone followed by a trough in levels by day 1. The high initial burst was attributed to a combination of the small particle size and high loading levels for both formulations. These results are in excellent agreement with previous studies that discuss the role of particle size and high drug loading on burst release [49, 54]. By day 4, levels rose slightly to release drug in a sustained manner with levels being depleted slowly through day 15. *In vivo* profile of the pharmacologically active metabolite, 9-hydroxyrisperidone, mimicked those of the parent molecule, albeit at slightly lower levels.

An initial burst was also observed with *Formulations C and D*, administered at a 40 mg/kg dose in rats. The highest burst was observed with *Formulation C*, which was prepared with the lower molecular weight 75 : 25 PLGA and had the smallest particle size, lowest bulk density value, and maximum drug loading, albeit the differences in these values are not significant. Aside from the initial burst, the profiles of *Formulations C and D* were similar. After an initial burst, a sharp drop occurred and the drug levels through day 22 remained in a steady manner while progressing to a decline up to day 45 for both formulations. In a manner similar to that observed with *Formulations A and B*, the metabolite levels were lower than Risperidone.

In summary, *Formulations A, B, and C* depict similar *in vivo* behavior that is characterized by a high initial burst, attributable to surface associated drug. Once initial burst was complete, depletion of circulating levels of drug led to a trough that was followed by a slow sustained release of drug from the PGLA matrix until values diminished. In contrast, mean plasma levels of Risperidone and its active metabolite, 9-hydroxyrisperidone, show a latency of nearly 3 weeks after administration of a single injection of Risperdal Consta in patients [27]. No initial burst is observed; rather, levels are low and almost flat till approximately 21 days after dosing, after which levels rise to peak at weeks 4-5 and last until week 7 leading to a slow decline in levels. This necessitates the intake of supplemental oral dosage forms for the first three weeks of the treatment regimen, making non-adherence to therapy a serious issue.

The initial burst phenomenon is an excellent platform for delivering a bolus dose. This type of effect is desirable in certain therapeutic regimens, especially those involving long term therapy. For instance, burst release of Leuprolide, a Luteinizing Hormone Releasing Hormone (LHRH) analog, from PLGA microspheres has been documented in literature reports [41, 42]. Leuprolide, a LHRH super-agonist, causes a spike in testosterone levels when administered after which testosterone levels drop to below chemical castration levels. Long acting injectables containing Leuprolide exhibit the initial burst phenomenon as it significantly impacts the pharmacodynamic effects *in vivo*. Similarly, for long acting injectable dosage forms of Risperidone, an initial burst is desirable. Ramifications of a lack of initial burst from the dosage form are that the schizophrenic patient will receive ineffective therapy, similar to the observed with the currently marketed formulation, thus necessitating the need for supplemental Risperidone.

#### 3.2.2. Cumulative AUC

Results of cumulative area under the curve (AUC) for the active moiety were calculated by the trapezoidal method (1), are shown in Table 2:

(1) $$\begin{array}{c} \text{AUC}\left(t_{1}-t_{2}\right)=\left[\frac{\left(C_{1}+C_{2}\right)}{2}\right]\times \left(t_{2}-\;t_{1}\right). \end{array}$$

In (1) “*t*” represents time in hours while “*C*” denotes serum concentration of Risperidone (ng/mL).

Results from AUC calculations indicate that the cumulative AUC values through 15 days for *Formulations A and B *were remarkably similar (1110 and 1159 ng × mL/day, respectively). Both formulations, administered at 20 mg/kg dose, were prepared using the fast degrading 50 : 50 PLGA copolymer had a small particle size and high loading but a difference of ~ 10 kDa in molecular weight. *In vivo*, they exhibited similar burst levels followed by a brief trough with noticeable levels through 15 days. Though the formulations exhibited a high initial burst, more than 98% of the cumulative AUC was contributed by drug encapsulated in the polymer matrix with initial burst amounting to a mere 1.4–1.8% of the total profile.

Cumulative AUC levels for *Formulations C and D*, dosed at 40 mg/kg, are presented in Table 2. Values of 1821 and 1522 ng × mL/day were obtained for *Formulations C and D*, respectively. As expected, values are higher than those observed with *Formulations A and B*. With *Formulation C*, initial burst contributed nearly 2% to the cumulative AUC whereas, with *Formulation D*, the value was smaller (1%). Once again, these data suggest that most of the *in vivo* activity was due to drug incorporated in the polymer matrix that was available for release in a sustained fashion.

In contrast, the marketed formulation does not exhibit initial burst and supplementation with oral therapy is needed to achieve pharmacologically effective levels of the drug [27], suggesting that drug encapsulated in the polymer matrix was solely responsible for *in vivo* activity.

The following observations were noted upon analyzing the cumulative AUC values of *Formulations A–D*.

- The contribution of initial burst towards the total AUC for all formulations was minor (equal to or less than 2%).
- Risperidone encapsulated in the PLGA polymer was responsible for over 98% of the cumulative AUC *in vivo*.
- The cumulative AUC obtained with *Formulations C and D* was nearly 1.5–1.7 times greater than that observed with *Formulations A and B*. These results suggest that proper choice of a copolymer and molecular weight will enable customization of drug release profiles from microsphere dosage forms of Risperidone.

#### 3.2.3. Selection of Dosing Regimen

The objective of the current study was to develop and evaluate PLGA microspheres of Risperidone that offered initial and maintenance levels of the drug for extended intervals. To predict the *in vivo* profile of Risperidone PLGA microspheres for a prolonged duration, plasma levels through 4 doses for all four formulations were simulated using the superposition principle. Simulations of multiple dosing have been used previously as they have excellent clinical utility due to the fact that they allow the medical professional to determine selection of an appropriate dosing regimen for a given formulation [55]. In addition, simulation experiments also provide information on the expected *in vivo* drug levels over an extended duration of treatment. Such types of studies are popular as they minimize the unnecessary usage of human and/or animal subjects in actual multiple dose pharmacokinetic studies and also offer time and cost savings to a clinician. Further, the multiple dosing simulations also provide data on the steady state concentration that are expected upon repeated dosing of a given formulation. Typically, simulation experiments require that concentration time data generated from a single dose be extrapolated to a multiple dosing scenario using the principle of superposition. Based on this principle, *Formulations A and B* with a short duration of action (Figure 2) would be dosed at different intervals from *Formulations C and D*.

**Figure 3:**
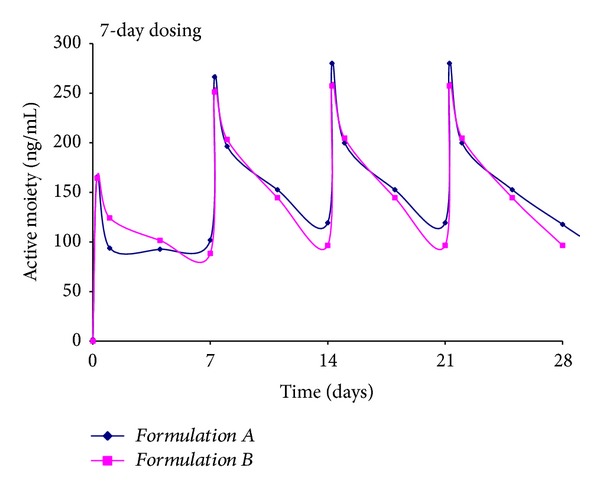
Simulation of multiple dosing regimen for *Formulations A and B *administered weekly, total = 4 doses.

Once a week dosing for *Formulations A and B* (Figure 3) shows active moiety levels between 100 and 260 ng/mL with an initial spike in drug levels observed after the administration of the first dose. As dosing continues, the peaks occur immediately after each administration but then fall quickly to 100 ng/mL only to repeat the peak and trough profiles throughout the 4 doses administered. In general, peak values of 280 ng/mL were obtained after dose 4 (steady state) with trough values of 100 ng/mL. Thus, *Formulations A and B* exhibited a pulsatile profile after simulations of multiple dosing. As expected from Figure 2, the similarity in behavior was attributed to the small particle size, high drug load, and high bulk density of the two formulations prepared using 50 : 50 PLGA.

**Figure 4:**
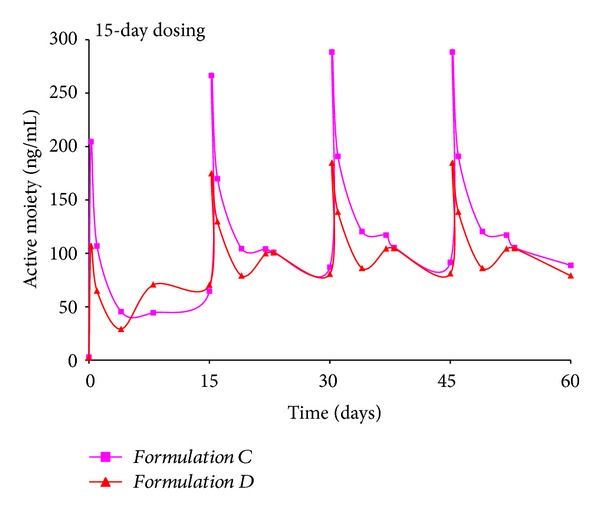
Simulation of multiple dosing regimen for *Formulations C and D *administered every 15 days, total = 4 doses.

For *Formulations C and D*, a 15-day dosing regimen was attempted (Figure 4). Once again, a pulsatile release profile is observed primarily due to the initial burst observed with both formulations. From an initial peak active moiety value of ~250 ng/mL for *Formulation C* and nearly 110 ng/mL for *Formulation D*, values reach 290 ng/mL for *Formulation C* and 190 ng/mL for *Formulation D*. The *in vivo* profiles of the two formulations are nearly similar, with the exception of the peak height of the initial spike. Throughout the course of dosing, active moiety levels ranged between 85 and 290 ng/mL and are similar to the range observed with *Formulations A and B*.

These results suggest that with the proper choice of PLGA polymer, similar blood levels can be obtained for different dosing regimens, that is, weekly or 15-day dosing. In fact, the pulsed behavior of these formulations also confirms that coadministration of Risperidone oral tablets is not necessary with all four formulations evaluated in this study. Further, selection of 50 : 50 and 75 : 25 polymers with appropriate molecular weight ensures that a significant portion of drug release has occurred from the microsphere prior to administration of the next dose. This is in complete contrast to the marketed preparation where the situation is completely reversed. Administration of the first dose of the marketed preparation shows minimal levels of Risperidone through 3 weeks with drug release occurring from week 4 to 7. Thus, even after administration of dose number 2, Risperidone levels *in vivo* will continue to be minimal. This suggests that when therapy is terminated, a longer washout period will be needed for patients dosed with the marketed preparation.

#### 3.2.4. Steady State

**Figure 5:**
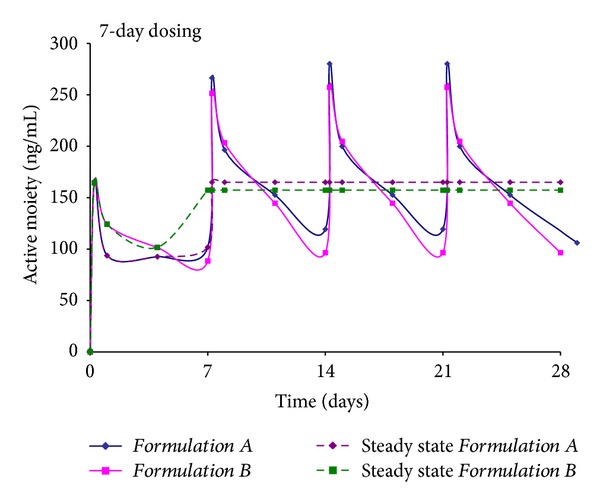
Average steady state concentration for *Formulations A and B. *

**Figure 6:**
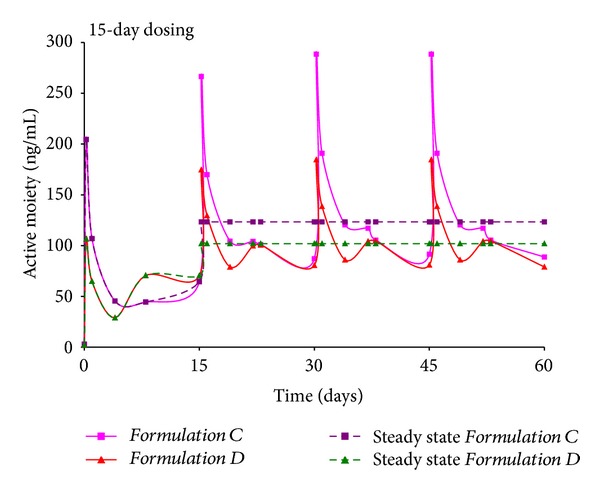
Average steady state concentration for *Formulations C and D. *

An important parameter that describes the *in vivo *performance of a formulation is its steady state concentration. In this study, steady state values for *Formulations A–D *were determined and are plotted in Figures 5 and 6. The average steady state concentrations for *Formulations A and B* were determined to be 165 and 157 ng/mL for weekly dosing of *Formulations A and B*. Based on the *in vivo* profiles obtained in rats, the similarity in steady state values was expected. A noteworthy observation is that steady state levels are achieved by the second dose, suggesting that Risperidone from *Formulations A and B* elicits its pharmacological actions rapidly, with no delay in response.

Similarly, steady state levels of 123 and 102 ng/mL were obtained for *Formulations C and D*, where dose of 40 mg/kg was administered every 15 days. Analogous to *Formulations A and B*, the steady state levels for the longer acting *Formulations C and D *were also similar. Slightly higher steady state levels were observed with *Formulations A and B*, prepared using the fast degrading 50 : 50 polymer, but overall, the values demonstrate consistency in *in vivo* drug release profiles over an extended interval.

The steady state levels for *Formulations A–D* reveal certain clinically relevant findings. Firstly, time to achieve steady state with the four formulations is short, that is, one week for *Formulations A-B* and two weeks for *Formulations C-D*. In comparison, given that marketed preparation shows minimal release for almost 3 weeks after administration, time to reach steady state is reported to occur after the 4th dose is administered [56]. Secondly, a spike in initial levels immediately after administration of doses 2, 3, and 4 allows for a bolus dose when drug levels from dose 1 taper off. In contrast, an oral tablet has to be administered to ensure a bolus dose with the current long acting injection. Finally, if patients on *Formulation A, B, C, or D* discontinue treatment, the washout period is small. With the marketed preparation, a 7-week duration of action implies a longer washout period, resulting in unnecessary exposure of Risperidone for the patient.

Indeed, results of this study confirm that utilization of PLGA polymers to encapsulate Risperidone allows both the researcher and the clinician to customize therapy for schizophrenic patients. Additionally, these dosage forms can eliminate patient compliance issues, minimize costs associated with therapy, and improve the quality of life for patients and caregivers. Hence, a proper choice of polymer properties to manufacture long acting injections of atypical antipsychotics shows great promise to efficiently and effectively treat patients suffering from schizophrenia.

## 4. Conclusions

The study demonstrated that sustained release microspheres of Risperidone utilizing two PLGA copolymers with varying lactide : glycolide ratios (50 : 50 and 75 : 25) as well as molecular weights had a strong potential to be excellent for providing initial and maintenance levels of Risperidone and its active metabolite. Results from the simulation study indicate that, when utilizing the superposition principle, simulations of weekly continual dosing of *Formulations A and B* and 15-day administration of *Formulations C and D* could be an effective approach for sustained delivery of this molecule and a possible alternative to the currently available combination therapy. Thus, proper selection of polymer properties to prepare long acting dosage forms with atypical antipsychotics will ensure patient compliance, reduce side effects, and improve the quality of life for patients who suffer from schizophrenia.

## Figures and Tables

**Table 1:** Properties of Risperidone PLGA microspheres.

Formulation	A	B	C	D
MW	45 kDa	74 kDa	54 kDa	65 kDa
PLGA type	50 : 50	50 : 50	75 : 25	75 : 25
Drug load (%)	25	34	34	33
Bulk density (g/cc)	0.76	0.67	0.65	0.68
Mean particle size (*μ*m)	24.6	18.9	17.1	21.9
Dose of Risperidone (mg/kg)	20	20	40	40

**Table 2:** AUC for Risperidone PLGA microspheres.

Formulation	A	B	C	D
Dose	20 mg/kg	20 mg/kg	40 mg/kg	40 mg/kg
Cumulative AUC (ng × mL/day)	1110	1159	1821	1522
